# Experience of organization of mental health monitoring in university clinic of Kharkiv national medical university

**DOI:** 10.1192/j.eurpsy.2021.843

**Published:** 2021-08-13

**Authors:** V. Korostiy, A. Maltsev, E. Kozmina, O. Platinuk

**Affiliations:** 1 Psychiatry, Narcology, Medical Psychology And Social Work, Kharkiv National Medical University, Kharkiv, Ukraine; 2 University Clinic, Kharkiv National Medical University, Kharkiv, Ukraine; 3 University Clinic, Харьковский национальный медицинский университет, Kharkiv, Ukraine

**Keywords:** telepsychiatry, university clinic, COVID-19, pandemy

## Abstract

**Introduction:**

The mental health care system in Ukraine is centralized and largely focuses on capacity for inpatient psychiatric treatment with 90% of funding allocated to inpatient psychiatric care at hospitals, much higher than countries who already have more decentralized care. Community-based mental health care options, including mental health provided at the primary health care level are currently limited or absent in the mental health system. Psychosocial support, as well as self-care and mental health promotion are also insufficiently developed. Covid-19 pandemic is serious challenge for health care system, especially for consultation liaison psychiatry.

**Objectives:**

Mental health monitoring and psychological support in University Clinic of Kharkiv National Medical University, Ukraine during COVID-19 epidemic.

**Methods:**

HADS, SCL-90, HDRS, HARS

**Results:**

During COVID-19 epidemic, implemented combination of off-line and eye-to-eye methods of mental health monitoring and psychological counselling for patients and medical staff in University clinic of Kharkiv National Medical University. Model of early detection and management of mental disorders based on multidisciplinary teamwork principles, сombination of off-line and eye-to-eye methods of screening, monitoring and psychological counselling for patients and medical staff. The online format proved beneficial because many patients of University clinic have trust issues and preferred not to deal with psychiatric services locally when it comes to mental health problems and it was accessible on epidemic conditions. An important part of the outreach work by the project was to destigmatize mental health problems.

**Conclusions:**

Combined model (off-line and eye-to-eye services) of mental health care is preferred compare to traditional approach in modern conditions.

